# Sense of School Belonging and School Reintegration for Students Hospitalized With Chronic or Complex Medical Diseases: Insights From a Grounded Theory Study

**DOI:** 10.1111/josh.70176

**Published:** 2026-06-16

**Authors:** Lucrezia Tomberli, Laura Vagnoli, Letizia Pavone, Elena Amore, Enrica Ciucci

**Affiliations:** ^1^ Department of Education, Languages, Intercultures, Literatures and Psychology (FORLILPSI) University of Florence Florence Italy; ^2^ Meyer Children's Hospital, IRCCS Florence Italy; ^3^ Independent Researcher Italy

**Keywords:** chronic illness, equity, hospitalization, inclusion, school belonging, school reintegration

## Abstract

**Background:**

A strong sense of school belonging (SoSB) supports students' emotional well‐being, engagement, and adjustment. For students hospitalized because of chronic or complex conditions, maintaining SoSB is challenging yet essential for successful school reintegration.

**Methods:**

Using a Grounded Theory approach, this study combined interviews with 16 parents, 32 mainstream teachers, 31 hospital teachers, and one association, alongside drawings from 14 hospitalized students. Data were collected pre‐ and post‐COVID‐19. Drawings were analyzed through a meaning‐making approach, and all materials underwent iterative coding and constant comparison.

**Results:**

Students reported strong emotional bonds with hospital teachers but felt forgotten by their mainstream schools. Peer contact was limited, and teachers felt unprepared to manage absences and reintegration. Structural gaps and weak communication between schools hindered continuity. Both formal (e.g., remote lessons) and informal (e.g., messages, drawings) exchanges, along with psychologists and associations, emerged as protective factors.

**Implications for School Health Policy, Practice, and Equity:**

Findings highlight the need for integrated school–health policies, teacher training, and structured collaboration to ensure equitable relational continuity.

**Conclusions:**

Promoting SoSB during hospitalization is crucial for recovery, resilience, and educational equity.

## 1 Background

Children and adolescents with chronic or complex diseases often experience prolonged hospitalization, disrupting daily routines and living apart from their homes, schools, and social environments. A biopsychosocial and ecological perspective highlights how illness affects development across physical, psychological, and social domains [1, 2]. Illness as a biographical disruption generates interconnected challenges, including medical burdens such as fatigue and treatment side effects, psychological vulnerabilities such as anxiety and reduced self‐efficacy, and social disruptions linked to separation from peers and school life [3, 4, 5, 6, 7, 8].

These conditions may lead to fear, isolation, and trauma [9, 10, 11, 12, 13], as well as impair academic performance and self‐esteem [7, 14, 15]. Children with medical conditions are also at higher risk of bullying, often exacerbated by stigma and limited peer awareness of the child's medical condition [16, 17]. Protective factors such as supportive peer relationships, effective school–home–hospital communication, and inclusive practices can buffer these risks. Teachers may likewise experience stress and burnout, particularly in the absence of adequate training on this matter and institutional support [18, 19, 20].

Maintaining school connection during hospitalization is crucial for students' well‐being and reintegration [21]. Hospital‐based schools aim to ensure educational continuity through individualized instruction adapted to students' medical conditions. Hospital teachers play a key role within these settings, providing individualized instruction, liaising with mainstream schools, and supporting students' emotional adjustment during hospitalization and school reintegration [22]. Within this context, the sense of school belonging (SoSB) represents a key construct. Defined as feeling valued and connected to the school environment, SoSB is associated with emotional well‐being, academic success, and social development [23, 24, 25].

Higher SoSB is linked to reduced distress and stronger relationships [26, 27, 28], whereas prolonged absence and limited communication may undermine sense of belonging [20, 29, 30, 31, 32].

Recent literature conceptualizes SoSB as a multifaceted construct encompassing relational, organizational, and technological dimensions [30, 33, 34], including peer and teacher relationships [35], participation in school life, continuity practices between hospital‐ and mainstream schools, and the use of digital technologies [20, 30]. Growing evidence identifies SoSB as a facilitator of well‐being and academic and psychological outcomes among students with chronic illness [32, 36, 37, 38, 39]. Alongside this focus, European child‐rights frameworks emphasize the inclusion of children's voices in research and practice, promoting narrative and meaning‐making methods to capture children's experiences within their broader social ecology [40, 41].

Despite extensive research on academic and emotional outcomes of pediatric illness [42], limited attention has been devoted to how hospitalized students maintain connections with their school communities, and few studies integrate children's voices alongside those of parents and teachers [43]. Addressing this gap is essential to understanding how school belonging is sustained during hospitalization and reintegration.

## 2 Aims of the Research

The aim of this research is to explore the connection between hospitalized students and their classmates, with a particular focus on understanding the role that *Sense of School Belonging* (SoSB) plays in the school experience of students with chronic medical diseases. To date, no studies have examined this aspect.

## 3 Methods

The study received ethical approval from institutional ethics committees overseeing the different phases of data collection. All procedures were conducted in accordance with approved protocols and ethical guidelines for research involving minors and vulnerable populations. Written informed consent was obtained from all adult participants and from parents or legal guardians of participating children, and assent was obtained from children in an age‐appropriate manner. Data were collected and stored in anonymized and aggregated form to ensure participants' confidentiality and privacy. Particular care was taken in handling sensitive materials, including children's drawings and narratives, to prevent identification.

This study adopted a grounded theory methodology, a qualitative approach suited to under‐explored phenomena and inductive conceptual development. Grounded theory guided sampling, data collection, and iterative coding procedures [44, 45, 46]. Semi‐structured and long interviews with parents and teachers were analyzed through grounded theory coding phases to identify recurrent patterns and higher‐order categories. To complement this process, children's drawings were examined using a meaning‐making approach, enabling access to children's subjective experiences in a developmentally appropriate way [40, 41, 42, 43, 44, 45, 46, 47, 48, 49, 50]. Five participant groups were involved: hospitalized students, parents, mainstream teachers, hospital teachers, and an association that promotes school inclusion. The association was included as its relevance emerged during early analysis and the evolving COVID‐19 context. Students and parents were interviewed only pre‐COVID, while mainstream and hospital teachers were interviewed both pre‐COVID (Time 1) and post‐lockdown (Time 2).

### 3.1 Participants

Fourteen hospitalized students (8 males, 6 females; ages 8–12, M = 9.93) were recruited from oncology (79%) and general pediatric (21%) wards. Sixteen parents (87.5% females) participated. Thirty‐two mainstream teachers and thirty‐one hospital schoolteachers took part across the two phases. One regional association focused on educational inclusion was involved post‐pandemic.

### 3.2 Research Tools and Procedure

Children produced two open‐ended drawings representing hospital‐based and mainstream school experiences. Drawings are widely used to explore children's emotions and meaning‐making around illness and schooling [51, 52, 53]. No content instructions or time limits were provided, and materials were freely chosen. Methodological rigor was maintained throughout data collection and analysis. Each drawing was independently examined by two researchers and discussed through interpretative triangulation to enhance analytical credibility [46, 54].

Parents participated in semi‐structured interviews, adapted to the hospital context, exploring school–hospital connections, school roles, educational continuity, and transition challenges.

Mainstream and hospital teachers and the association took part in long‐form interviews. Pre‐COVID teachers were randomly selected, while post‐COVID participants were voluntary. Hospital teachers were identified through regional mapping of hospital education services. The association was identified through a systematic mapping of regional organizations, with the identification independently verified by the research team.

**FIGURE 1:**
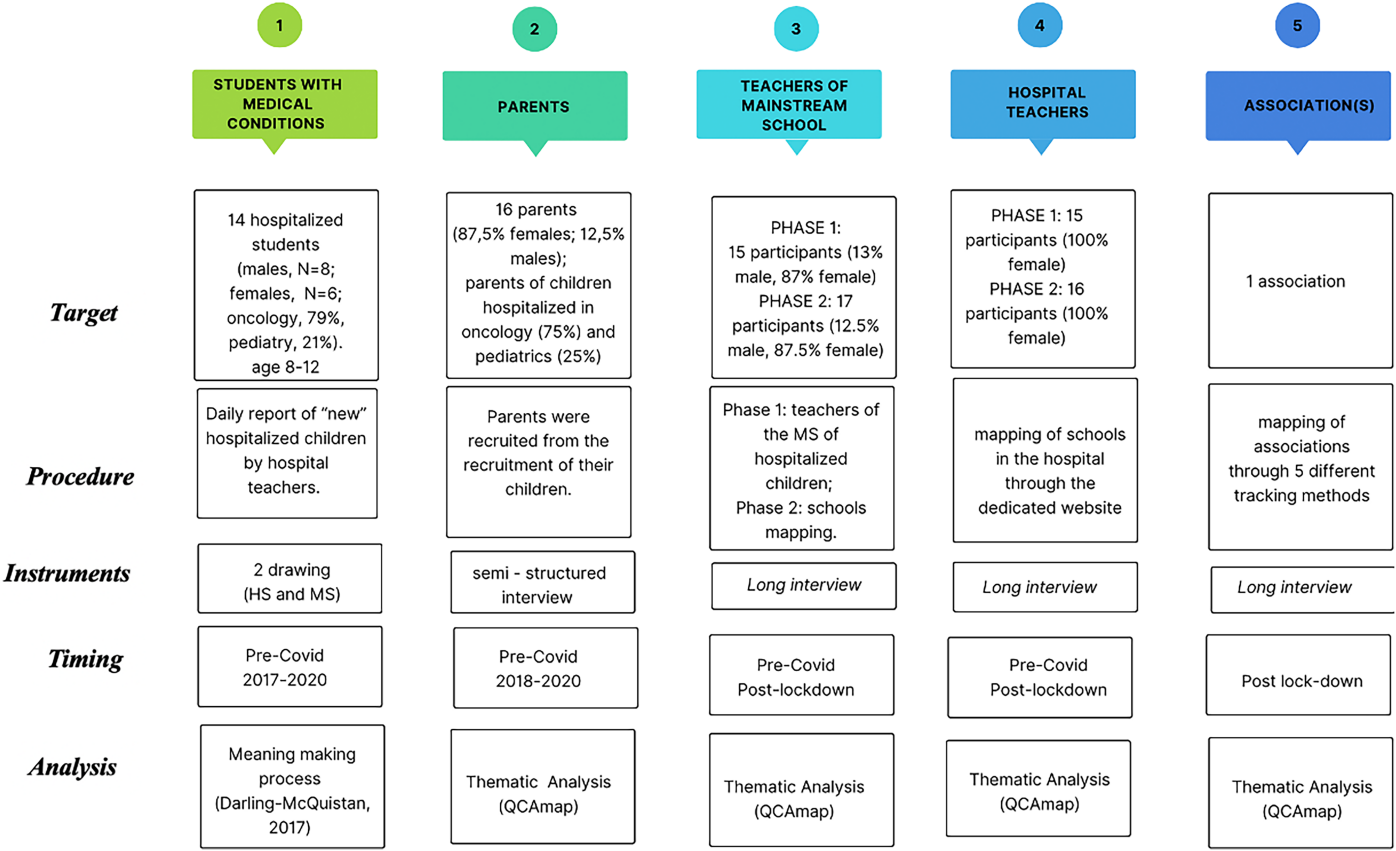
Summary of the research methodology.

The overall methodological framework of the study, including participant groups, recruitment procedures, instruments, timing of data collection, and analytic approach, is summarized in Figure 1.

### 3.3 Data Analysis

Researchers first conducted a descriptive analysis of the drawings to identify similarities and differences. This analysis was further developed using a meaning‐making approach, in which children were invited to explain their drawings and their narratives were recorded [48, 55]. Researchers used open‐ended questions to encourage children's free expression and elaboration [56]. Consistent with this approach, the analysis privileged children's subjective interpretations rather than external adult readings [49].

For the semi‐structured and long interviews, data were analyzed using grounded theory coding procedures (open, axial, and selective coding) [46], supported by QCAMAP software. Consistent with grounded theory principles, categories emerged through an inductive constant comparative process [57, 58]. Both drawing and interview analyses were embedded within the same grounded theory framework. To enhance trustworthiness, at least two researchers independently coded the material and discussed interpretations through researcher triangulation [54].

## 4 Results

Illustrative participant quotes supporting each emerging category are reported in Table 1.

**TABLE 1:** Illustrative quotes linked to emerging categories.

Theme/category	Participant	Illustrative quote
Hospital school as opportunity to future orientation	Hospital teacher	*“Hospital school helps the child to think about the future, to imagine what will come after the hospitalization. The hospital is like a big parenthesis in life, everything there feels so different from the outside: medical visits, clowns, animals, procedures. But hospital school gives them the chance to do something normal, to look ahead”*
Hospital school as normalcy	Hospital teacher	*“Hospital school is not just about keeping up with lessons—in fact, that's only a very small part of it. Hospital school helps the child feel normal, like everyone else, and look beyond the ward, toward everyday life. Through school we work on what is waiting for the child outside”*
Promotion of autonomy and emotional separation from parents	Parent	*“For us, school was miraculous. Our son managed to gain independence and autonomy; he really changed while attending hospital school. I also deeply value this service because it is finally something where we, as parents, step aside. It's good for our well‐being—we can even take time for a coffee—and it's good for the children too, because they need moments without a parent always around. And in that space, the teachers are there for them”*
Reframing the meaning of school	Parent	*“Before Giacomo's hospitalization (a pseudonym), I always thought school was about studying, becoming educated, getting good grades, and securing a good future. Here, I realized it is much more: an opportunity for growth, a way of being with oneself and with others, a chance to develop values… I carry this with me even now that Giacomo is in day hospital and has returned to his school—I no longer look at school the way I did before the hospitalization”*
Lack of awareness of hospital school services	Mainstream teacher	*“I had no idea this service even existed before finding myself with a child with cancer in school and discovering from other parents that something like this was available. I felt completely unprepared”*
Lack of communication and institutional support	Mainstream teacher	*“We have no training on the subject, no one tells us what to do, and the school gives us no guidance. We receive no information from the principal either. Everything is managed internally by the hospital, and often there is no way to have clear communication with them. Yes, there is some coordination between the hospital and the school coordinator, of course… but this does not involve the whole teaching team, because at middle school we are so many, and aligning everyone's schedules is practically impossible. I felt alone”*
Feeling forgotten by mainstream schools	Hospital teacher	*“It is very frustrating to have to manage everything on our own, and unfortunately this happens very often. The school seems to forget about the students who are absent from the morning roll call. Here we have children and adolescents who stay away from school for weeks, sometimes even months… and never once do the teachers call us. I ask myself: how is this possible? Don't you think of asking how they are, what they are doing, how you might be useful? Surely we have a serious problem of communication, and at times I fear also of sensitivity and humanity.”*
Feeling forgotten by mainstream schools	Hospital teacher	*“What saddens me most for our children is that they themselves feel forgotten. Teachers seem completely uninterested in the matter, as if after hospitalization the child no longer concerned them… yet they are still part of their class, even at a distance”*
Teacher uncertainty and fear of intrusiveness	Mainstream teacher	*“We don't know how to behave with these children who are no longer there. We don't see them for months, sometimes the parents don't say anything, taken up as they are by the illness—which, as a teacher and also as a parent, I understand… but I am still in difficulty because I would feel intrusive if I asked, and the result is that often we don't do anything. When we were told that Vanessa would return to class, I panicked. Luckily the school psychologist was there to help us… otherwise we would have made quite a mess.”*
Importance of supportive school relationships	Hospital teacher	*“You can clearly see when the relationship with the school is good or not. If it is good, the child is calm, feels thought of by classmates and teachers, and feels less alone… sometimes they even study more! But above all, what I always try to make schools understand is that a child who feels abandoned will not want to return to school, whereas one who misses their classmates and feels that they are being thought of will be more willing to return to their school niche, to their classroom made of painted walls and shared memories”*
Emotional bond with hospital teachers	Parent	*“In the hospital a unique relationship with the teacher is created. I don't know what we would have done without the teacher… here teachers dedicate themselves completely to children, because there is truly a one‐to‐one relationship or at most a very small group… and they get to know them so well. My son always tells me: ‘Mom, when we go back home, can we go visit the hospital teacher?’”*
Emotional bond with hospital teachers	Hospital teacher	*“I have many years of experience, both as a mainstream teacher and as a support teacher, and for some years now I have been working here in the hospital school… and I can state without a shadow of a doubt that here a special relationship is created, in some ways similar to that between a student and a support teacher in the outside school.”*
Differences between hospital and mainstream teaching methods	Hospital teacher	*“You cannot think of teaching Literature here the way you would in a regular classroom. Imagine having a child who is vomiting all the time because they are undergoing chemotherapy… how can you explain Dante in front of a blackboard in that situation? It's clear that hospital schooling cannot be done in the same way, and I would even say it's right that it shouldn't! Here we use different methods, tailored for each child. Every time we need to create something new and invent a highly personalized teaching approach, designed around the individual student. The primary school colleagues often manage this even better, because they spend more hours with the children. We are there for fewer hours and there are more of us teachers, so we know the students less well; some have only short hospital stays… But teaching here is truly a creative job. I compare it to being a painter: you know you have your colors, your techniques, your palette, you know you want to paint a beautiful landscape—but you have to figure out which one, with what light, with what technique, what shades… and then maybe the final result is a mess, and you have to start again, inventing something new once more.”*
Differences between hospital and mainstream teaching methods	Mainstream teacher	*“We do not agree with the methods used in the hospital because they are too far from the reality of mainstream schooling. I understand that in the hospital there is time to do wonderful things and to dedicate an extraordinary amount of time to each student, but the reality of school is different… and then the students no longer know how to study normally from books, because they confuse studying with playing. Perhaps we should find an approach that is a bit closer to what we normally do outside, or at least work out a middle ground together.”*
Limits of group activities in hospital school	Hospital teacher	*“Here in the hospital we try to do activities in groups, but it is very difficult… the classroom is designed to host several children, so we can do multiple things in parallel, but it is not like a real class group where everyone does everything together. At most, we manage to organize workshops, which in my opinion are truly a wonderful experience for the children, but even this is very different from regular school because the children here are constantly changing. They often cannot build friendships with each other, except for those in certain wards who stay longer. They are really two very different environments.”*
Role of psychological support in reintegration	Mainstream teacher	*“The school psychologist was essential during Mattia's hospitalization. Without her we would not have been able to do anything… she helped us from the very beginning, telling us how to balance the family's privacy while still showing interest… and then how to welcome him back into class without making him feel strange or different… The psychologist was also in contact with the hospital school and took part in team meetings, and this helped a lot”*
Fragmentation of psychological services	Association member	*“Unfortunately, psychological services are fragmented. We collaborate well with the hospital's doctors and psychologists, and in the most collaborative cases we manage to establish a good connection with the mainstream school as well… but sometimes this is not enough, because without sufficient psychologists in schools, teachers do not know what to do and we cannot reach every school. And when we cannot, sometimes it is assumed that at some point the child simply goes back to school as before—but it is not like that. Returning to school must be imagined, dreamed of, planned, built… it has to be done in synergy. Otherwise, it is like delivering a post package.”*
Limits of distance learning	Hospital teacher	*“For a while, with distance learning, some even thought that the hospital school was no longer necessary… but then it became clear that this is not the case, because we cannot be replaced by a computer. It is wonderful that children can connect with their school and attend lessons online—this is actually something we had always tried to promote even before the pandemic… but there still needs to be a human element that makes the child feel that the school is truly with them, and not just on a computer screen.”*
Impact of COVID‐19 on inclusion practices	Hospital teacher	*“The pandemic was obviously a very critical period here in the hospital. We were in full emergency, we could not do anything, we were completely kept out of the wards, we could not approach the children, especially those in oncology or other complex wards… wards where children already feel lonely, and we had to leave them even more alone… We tried to compensate with distance learning, but it was not the same. Some mainstream schools had the idea—or together we came up with the idea—of also inviting hospitalized children to take part in distance learning… other schools did not think of it at all, but on the other hand, if you don't think about inclusion before the pandemic, you certainly don't think about it in an emergency.”*

### 4.1 Emotions in Participation During Hospital School Activities

The data highlighted how the hospital school (HBS) can foster future orientation, provide a sense of normalcy, act as a distraction from illness, and maintain connections with the student's mainstream school (MS). Overall, HBS was described by parents and teachers as promoting continuity in education and facilitating smoother school reintegration. Hospital teachers emphasized that HBS helps students feel less isolated and more “*equal to their peers*,” while supporting healthy aspects of identity development through everyday activities, personal growth, and life skills beyond academic learning. HBS was also reported to promote children's autonomy and emotional separation from parents during hospitalization, helping preserve their identity as students. Parents considered this particularly important given the regression in independence often associated with illness, whereas teachers highlighted the central role of collaboration between hospital, school, and family in sustaining school belonging.

Finally, participation in HBS contributed to a broader reframing of the meaning of school among both parents and teachers. School was increasingly viewed not only as a place for academic achievement but also as a relational and developmental space, with parents experiencing emotional relief and teachers recognizing the importance of relationships alongside learning.

### 4.2 Relationship Between Hospital School and Mainstream School

Mainstream teachers described communication with the HBS as largely mediated by hospital school coordinators or support services, which often generated feelings of distance and lack of communication and institutional support, particularly at the secondary school level where teacher–student contact is more limited. This delegation contributed to teachers' experiences of isolation and reduced involvement in supporting hospitalized students.

In contrast, hospital teachers perceived limited engagement from mainstream teachers in maintaining school–hospital connections, with some reporting that hospitalized students were sometimes forgotten by mainstream schools, especially when staff changes occurred. Both groups identified a shared challenge in the absence of adequate training to manage school–hospital coordination and foster school belonging.

These relational dynamics highlighted the importance of supportive school relationships in sustaining connection and reintegration. Several teachers described uncertainty and fear of intrusiveness, which often resulted in inaction when a student was hospitalized. In this context, school psychologists emerged as key figures in guiding teachers and facilitating reintegration processes, reinforcing the idea that students remain active members of their school communities throughout hospitalization.

### 4.3 Teacher‐Student Attachment

The findings showed that the relationship between students and hospital schoolteachers differs substantially from that in mainstream schools, primarily due to the one‐to‐one interaction characteristic of the hospital setting. While mainstream teachers typically engage with entire classes and have fewer opportunities for dyadic relationships, the hospital context appears to facilitate the development of a strong emotional bond with hospital teachers across all participant groups.

**FIGURE 2:**
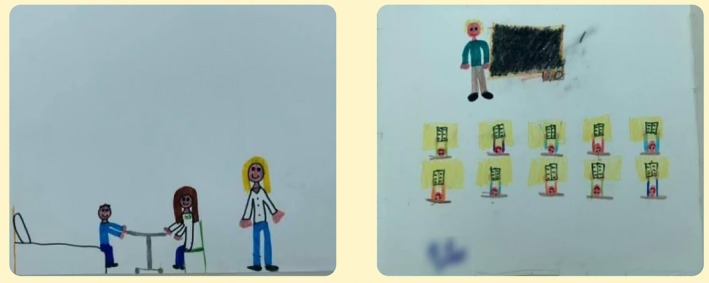
Children's representations of hospital‐based and mainstream school contexts: dyadic teacher–student interaction versus collective classroom environment.

Children consistently depicted their hospital schoolteachers in their drawings and described close, positive relational experiences, which were also confirmed by parents and hospital teachers. Notably, drawings of the hospital school context focused exclusively on the dyadic teacher–student relationship, with no peers represented. This pattern is illustrated in Figure 2, where the hospital school is portrayed as a one‐to‐one interaction beside the hospital bed. In contrast, drawings of mainstream schools often emphasized the physical classroom environment, such as desks and blackboards, and sometimes omitted both teachers and classmates, suggesting a more impersonal representation of the school context.

### 4.4 Sense of School Belonging and School Reintegration: Organizational and Relational Issues

Overall, the findings highlighted significant organizational differences between hospital schools and mainstream schools that may hinder reintegration processes. Hospital teachers emphasized that the specific temporal and spatial constraints of the hospital context require tailored teaching approaches, while mainstream teachers perceived differences between hospital and mainstream teaching methods as creating a disconnect in students' educational experiences and complicating their return to school.

Beyond organizational aspects, the relational dimension emerged as central for students' emotional experiences during hospitalization. Children's drawings and interviews consistently portrayed the HBS as a highly relational context, primarily focused on the dyadic teacher–student relationship rather than on peer interactions. This perception aligned with reports from parents and teachers, who highlighted the role of HBS in fostering feelings of normalcy, equality, and emotional support.

At the same time, the hospital context posed major challenges for implementing collective learning experiences. Limits of group activities in hospital school were reported by hospital teachers, who explained that differences in students' ages, curricula, and length of hospitalization made sustained group teaching difficult, in contrast to mainstream schools where classes share long‐term peer relationships and collective experiences.

**FIGURE 3:**
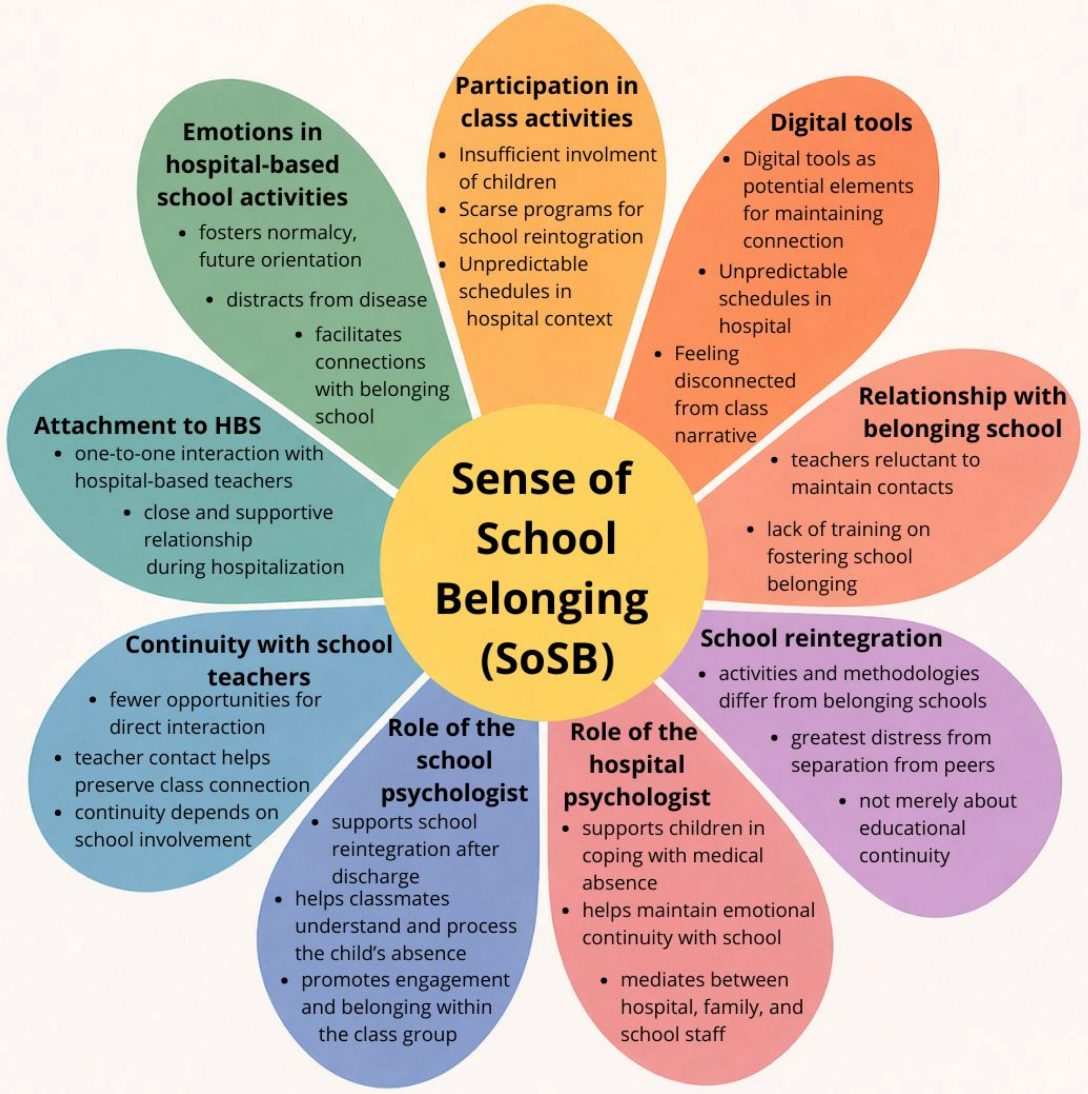
Dimensions of school belonging during hospitalization.

In children's drawings, mainstream schools were strongly associated with peer relationships, including both formal classroom moments and informal social interactions. Some children avoided drawing their mainstream school, describing sadness for missing classmates. This experience was echoed by parents and teachers, who reported that hospitalized students often felt forgotten by their schoolmates. Overall, maintaining educational continuity appeared insufficient to sustain a strong sense of school belonging. Figure 3 provides a visual synthesis of the main dimensions of school belonging that emerged from the analysis, highlighting how relational continuity, teacher–student attachment, participation in class activities, psychological support, and digital tools interact in shaping hospitalized students' school experience.

### 4.5 Need for Specific Training for School Staff

The findings revealed substantial gaps in the training of mainstream teachers, who often reported feeling unprepared to manage the complexities of students' illnesses, promote inclusion, and prevent school dropout during medical absence. These difficulties were confirmed by hospital teachers, parents, and students, with children's drawings and narratives reflecting emotional distress and feelings of disconnection from their schools. Parents particularly described challenges in maintaining communication with teachers, especially at the middle school level, while some students avoided drawing their mainstream school due to sadness linked to feeling forgotten.

Despite these challenges, not all mainstream teachers perceived additional training as necessary, arguing that existing competencies in special pedagogy were sufficient. However, these same teachers described difficulties when facing unexpected situations, including parental resistance to maintaining connection, school phobia during reintegration, communication with classmates about illness, managing peers' fears, and coordinating with hospital services.

In contrast, hospital teachers strongly emphasized the importance of specialized training, although many reported they learn primarily through experience rather than formal preparation. While professional development opportunities have increased over time, training remains non‐mandatory and most effective when delivered by professionals with in‐depth knowledge of the HBS context.

Finally, the involved association highlighted the central role of teacher training and network‐building among school, family, and hospital in fostering school belonging. Although the association partially addressed training gaps, the study identified only one local association specifically dedicated to preparing teachers and raising awareness about school inclusion for students with medical conditions.

### 4.6 The Role of the Psychologist in Promoting School Inclusion and Sense of Belonging

Both hospital and mainstream teachers emphasized the central role of psychological support in reintegration, particularly during students' return to school, which was described as one of the most challenging phases of the illness trajectory. Prolonged absences often led hospitalized students to feel disconnected from their classroom communities, undermining their sense of school belonging.

Teachers also reported difficulties in maintaining structured relationships between hospitalized students and their classmates, especially in secondary school, where this responsibility was often left to informal peer interactions. The absence of organized programs supporting reintegration in both formal and informal school contexts further intensified these challenges.

At the same time, teachers expressed concerns about the feasibility of involving psychologists in managing communication about a classmate's illness. Information was frequently shared spontaneously through informal channels such as informal conversations or messaging apps, making it difficult to control or structure. However, teachers acknowledged that while initial communication may be hard to formalize, the subsequent phase of helping students process and understand the situation could be addressed more systematically.

In contrast, the involved association emphasized that structured support across the entire disease trajectory is achievable, provided that effective collaboration and networking between school, family, and hospital are established. From this perspective, current difficulties were linked to the fragmentation of psychological services, which limited continuity of support.

### 4.7 Sense of School Belonging and Participation in Class Activities

The findings highlighted that a strong sense of school belonging requires students' active participation in school life, involvement in the class narrative, and engagement with the symbolic elements of the school environment. When these components are lacking, hospitalized students risk feeling abandoned, misunderstood, and emotionally disconnected from their schools.

Hospitalized students were often insufficiently involved in class activities during prolonged absences, threatening their sense of belonging. Although mainstream teachers frequently reported “being in contact” with hospitalized students, closer examination revealed that communication was primarily maintained with parents or hospital teachers rather than directly with students. This pattern was confirmed by parents and hospital schoolteachers, who described persistent difficulties in sustaining regular and informal school–hospital connections.

When peer contact occurred, it was often spontaneous and student‐started. While some teachers viewed this positively as promoting autonomy and peer relationships, others emphasized that reliance on informal communication frequently resulted in prolonged disengagement and weakened sense of school belonging. Direct contact with hospitalized students was generally infrequent and mainly focused on academic matters rather than relational support.

Hospital teachers further noted that mainstream schools often struggled to address students' relational needs alongside educational plans. Although positive reintegration experiences were reported in some cases, the findings suggested that many students, despite feeling welcomed upon return, did not fully regain their place within the ongoing class narrative they had been part of prior to hospitalization.

### 4.8 Digital Tools to Promote a Sense of School Belonging: Balancing Opportunities and Challenges

All participants emphasized that technology has long been considered essential for maintaining the school–hospital connection, both educationally and relationally. Before the COVID‐19 pandemic, however, mainstream schools and the association had not implemented online educational activities and often perceived such connections as impractical, whereas hospital teachers already believed continuous distance learning was feasible.

In the pre‐pandemic phase, difficulties were mainly attributed to poor internet connectivity and limited digital skills. Although these technical barriers were partially resolved during the pandemic, new and more complex challenges emerged. Teachers reported difficulties integrating hospitalized students into distance and hybrid learning due to medical routines, limited attention in online settings, and challenges in sustaining active participation.

The pandemic experience also revealed the emotional and professional burden on teachers, reducing time and resources for maintaining school–hospital connections. Importantly, it highlighted the irreplaceable role of face‐to‐face presence and meaningful human relationships in supporting hospitalized students. While remote lessons were once considered sufficient to maintain connection, post‐pandemic teachers no longer viewed technology alone as adequate for fostering engagement and a sense of school belonging.

Overall, although COVID‐19 accelerated the adoption of digital tools in education, it did not substantially improve inclusion for hospitalized students, who continued to experience isolation as well as peers affected by lockdowns.

### 4.9 Individual and Relational Emotional Experiences Related to the Pandemic

Teachers reported that some students initially experienced a sense of normalcy during the pandemic, feeling part of a shared experience of isolation. Over time, however, students became increasingly aware of the difference between hospitalization due to illness and lockdown‐related isolation, which heightened feelings of difference and exclusion.

Distance learning reinforced misconceptions among mainstream teachers, who sometimes assumed that HBS was no longer necessary, reflecting the perceived limits of distance learning for maintaining meaningful school–hospital connections. In practice, hospitalized students were included under the same conditions as peers in quarantine, without consideration of their distinct routines and needs, resulting in minimal participation and increased disconnection.

Hospital teachers also described a weakening of student–teacher relationships during remote lessons, particularly in demanding wards, contributing to lower mood and emotional distress among hospitalized children. These experiences reflected the broader impact of COVID‐19 on inclusion practices within hospital education. One positive change involved classmates' parents, whose resistance to school–hospital contact decreased after the pandemic, with greater openness to communication and recorded lessons to support inclusion.

## 5 Discussion

The present study underscores the central role of hospital schools in fostering normalcy, future orientation, and educational continuity for hospitalized students. Consistent with research showing that hospital schooling mitigates educational disruption by reinforcing children's identity as learners rather than patients [59, 60], our findings demonstrate that HBS functions as a symbolic bridge toward life beyond hospitalization. This aligns with family‐centered interventions emphasizing educational identity [61] and qualitative studies highlighting school as a domain for envisioning a future beyond illness [32].

HBS also promoted personal growth and emotional autonomy through close dyadic teacher–student relationships. In line with attachment‐oriented perspectives, these bonds fostered resilience in medicalized environments [62, 63].

Despite recognition of school belonging, fragmentation between hospital and mainstream schools emerged. Limited awareness of HBS, institutional isolation, and insufficient training weakened collaboration. These findings confirm European evidence of systemic disconnection [64] and Italian research showing that pandemic‐driven digital innovation did not resolve relational gaps [64, 65]. Fragmentation appeared both structural and relational, reflecting limited institutional recognition of hospital schooling.

A key contribution concerns the redefinition of school belonging in hospital contexts. While belonging in mainstream schooling is largely grounded in peer relationships [66, 67], hospitalized students primarily experienced belonging through adult–child dyads with hospital teachers, consistent with individualized pedagogy [68]. Nevertheless, peer connections with the original class community remained crucial, sustaining social identity [69], supporting academic motivation [70], and facilitating school reintegration [33, 71]. When continuity was lacking, students experienced isolation and anxiety about returning to school [32].

These findings invite a shift in perspective, positioning the mainstream school as the student's *school of belonging*, rather than simply the place to which they return. From this perspective, students remain part of their school community throughout hospitalization, supporting meaningful inclusion and a smoother reintegration process, in line with recent work on school reentry pathways [30].

Organizational differences between HBS and mainstream schools further complicated reintegration. While HBS emphasizes individualized approaches [68], MS operate within collective classroom structures and standardized curricula, creating barriers to coordination. Although digital technologies were viewed as potential bridges, their effectiveness proved limited when used alone. Post‐pandemic challenges included medical routines, reduced online engagement, and professional fatigue. These findings support concerns regarding digital‐only solutions [38]. While innovative tools have been explored [72, 73, 74], they remain exploratory. A recent Delphi study suggests that flexible asynchronous tools may offer more balanced approaches [30].

Psychological support emerged as essential during reintegration, aligning with interdisciplinary and systemic models [30, 75, 76].

Overall, school belonging emerged as a multidimensional construct encompassing academic, relational, and identity‐related processes [33], requiring deliberate support beyond academic continuity.

### 5.1 Limits of the Study

This study has several limitations that should be considered when interpreting the findings. First, the qualitative sample was relatively small and did not include post‐pandemic student participants, limiting the possibility of capturing changes in children's experiences over time. In addition, findings may be context‐specific and not fully transferable to other educational or healthcare systems, particularly given the organizational features of hospital schooling in the European context. The study also relies more extensively on the perspectives of adult informants (parents and teachers), which may have shaped the interpretation of children's experiences despite the inclusion of drawings and narrative accounts.

Nevertheless, the integration of multiple perspectives, the use of complementary qualitative methods, and the application of grounded theory procedures support the robustness of the findings. Moreover, the alignment with belonging theory and child‐rights frameworks [40, 41] provides a coherent interpretative lens that recognizes hospitalized children as active participants in their educational and developmental trajectories.

### 5.2 Implications for School Health Policy, Practice, and Equity

This study highlights implications for school health professionals supporting students with complex medical conditions. Maintaining hospitalized students' inclusion within the class experiences and school community is essential to prevent psychological distress during school reintegration. Many students felt forgotten when there was no informal connection with schoolmates and mainstream teachers, even when educational continuity was ensured.

To foster SoSB, collaboration between hospital and mainstream schools should promote both formal connections (e.g., remote lessons) and informal interactions (e.g., messages, peer initiatives). Active participation is necessary, and mainstream teachers should be supported in maintaining meaningful contact.

Targeted training for teachers and staff is crucial, as many felt unprepared to address students' medical and emotional needs. Training should integrate didactic and relational competencies and should be developed with hospital teachers and healthcare professionals.

Psychologists play a central role in communication, emotional support, and reintegration planning and should be systematically involved. External associations can further assist through tailored interventions and awareness initiatives about illness and school reintegration.

While digital tools can facilitate school–hospital connections, they cannot replace relational engagement and must be embedded within meaningful human relationships.

Overall, promoting school belonging requires investment in teacher training, inclusive practices, psychological support, sustained school–hospital collaboration, and clear policy guidelines.

## 6 Conclusions

Hospitalization disrupts students' connection with their school communities, placing their sense of school belonging at risk. This study highlights the need to reconceptualize belonging in hospital contexts as relational, organizational, and identity‐based. Supporting school belonging requires coordinated efforts across hospital and mainstream schools, integrating educational, relational, and psychological dimensions to ensure continuity, inclusion, and equity in students' educational trajectories.

## Funding

This work was supported by University of Florence (Italy) with a research grant.

## Ethics Statement

This study is part of a broader project approved by two ethical committees:

1. pre‐Covid 19 data collection (involving children and parents) was approved by Meyer Pediatric Ethical Committee (n. of register: 08/2019, emendamento n. 4 of 03/01/2019, Prot. Scuola in Ospedale)
2. the second data collection after pandemic was approved by FORLILPSI Department of University of Florence Ethical Commettee (n. SIO_Unifi, parere 138 del 05/02/2021).

As per the protocol submitted to the ethics committee, each research participant signed an informed consent form to take part in the present study. The informed consent form was prepared and approved by the aforementioned ethics committees. The research data are stored in an anonymized and aggregated manner, in accordance with the informed consent and the research protocol forms approved by the ethics committee, to ensure participants' privacy. Further information is available in the records of the ethics committees (in Italian).

## Conflicts of Interest

The authors declare no conflicts of interest.

## Data Availability

The data that support the findings of this study are available from Meyer hospital (privacy of patients and participants). Restrictions apply to the availability of these data, which were used under license for this study. Data are available from the author(s) with the permission of Meyer hospital (privacy of patients and participants).
